# Benign Intranodal Thyroid Tissue Similar to Nodal Metastasis of Thyroid Papillary Carcinoma: A Rare Case Report

**DOI:** 10.3390/diagnostics13030577

**Published:** 2023-02-03

**Authors:** Yoo-Na Kang, Jung-Guen Cha

**Affiliations:** 1 Department of Forensic Medicine, School of Medicine, Kyungpook National University, 680 Guk-chaebosang-ro, Jung-gu, Daegu 41944, Republic of Korea; 2 Department of Radiology, School of Medicine, Kyungpook National University, 680 Gukchaebosang-ro, Jung-gu, Daegu 41944, Republic of Korea

**Keywords:** intranodal, thyroid gland, papillary carcinoma

## Abstract

In patients with thyroid nodules, if the cervical lymph nodes gradually enlarge, a histological confirmation is required to rule out malignancy. Here is a case of benign intranodal thyroid tissue with cystic changes resembling lymph node metastasis of a papillary thyroid carcinoma. A 47-year-old man received ethanol sclerotherapy because of repeated enlargement of the thyroid gland 2 years prior to presentation. Subsequently, the patient underwent abscess removal from the deep neck and partial lobectomy of the attached left thyroid gland. Two months before the visit, extensive cervical lymphadenopathy was detected on ultrasonography (US) and computed tomography (CT). Total thyroidectomy and cervical lymph node dissection were performed to differentiate between metastatic papillary carcinoma of the thyroid gland and benign thyroid inclusions. Microscopic examination revealed multiple variable-sized nodules of benign thyroid follicles with cystic changes in both thyroid glands and bilateral cervical lymph nodes. An occult papillary microcarcinoma strongly positive for HBME-1 was also observed in the left thyroid lobe. However, the benign intranodal thyroid tissue was negative in both the real-time PCR-based BRAF V600E mutation test and HBME-1 immunohistochemical stain. Similarly, benign intranodal thyroid tissue can be enlarged by multiple cystic changes in a large number of lymph nodes along the neck node chain. For the differentiation of metastatic thyroid papillary carcinoma, real-time PCR-based BRAF V600E mutation test and HBME-1 immunohistochemical staining in addition to histological examination are helpful.

**Figure 1:**
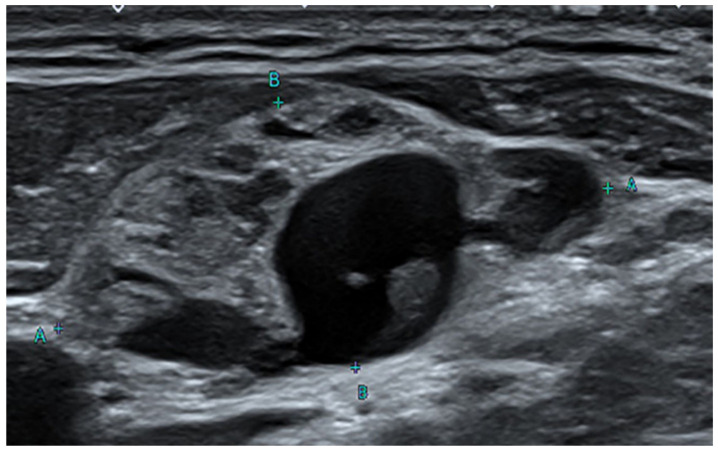
Ultrasound sonography (US) of the neck. Neck US reveals an enlarged lymph node with internal cystic change at left level III. A few cases of non-malignant inclusions were detected in lymph nodes removed from a patient with malignancies. For example, benign salivary glands in the pulmonary hilar lymph node, benign epithelial tissue, nevus cells in the axillary node, and benign Mullerian inclusions in the pelvic and paraaortic lymph nodes have been found [1,2,3,4,5]. There have been many reports since the first report of an intranodal inclusion of benign thyroid tissue in 1897, but the origin and mechanism of intranodal thyroid tissue are not clear [6,7,8,9,10]. Furthermore, there have been few radiographic pathological reports of very extensive cystic changes in benign thyroid tissue in the lymph nodes. A 47-year-old male patient visited the hospital 3 years ago complaining of discomfort in the left side of the neck. Ultrasound sonography (US) revealed thyroid nodules of various sizes with bilateral cystic changes. The thyroid function test was normal with T3 0.66 ng/mL, Free T4 1.36 ng/dL, and TSH 1.48 uIU/mL. Fine needle aspiration was performed to confirm nodular hyperplasia of the thyroid gland. Three months later, the size of the thyroid gland had increased. Therefore, direct injection of ethanol through the needle for the atrophy of a thyroid mass, the so-called ethanol sclerotherapy [11], was performed. The following day, he had trouble breathing with fever, chills, and myalgia. Computed tomography (CT) of the neck revealed a purulent cystic mass with conglomerated lymph nodes in the left neck. WBC 14,530/µL, ESR 120 mm/h, and CRP 39 mg/L were observed in the blood tests. A purulent abscess was clinically suspected and subtotal left lobectomy was performed along with abscess removal. An abscess of the neck and nodular hyperplasia of the thyroid gland were confirmed histologically. Four months later, a mediastinal mass was detected, and an invasive thymoma was diagnosed via total thymectomy. US of the neck performed 2 months prior showed enlarged lymph nodes at left neck levels II and III, and increased blood flow in the solid part (Figure 1). Polymerase chain reaction (PCR) test for Mycobacterium tuberculosis was negative, and the BRAF V600E mutation was not detected. CT of the neck revealed multiple nodular lymph node masses from left neck levels II, III, and IV to the superior mediastinum (Figure 2).

**Figure 2:**
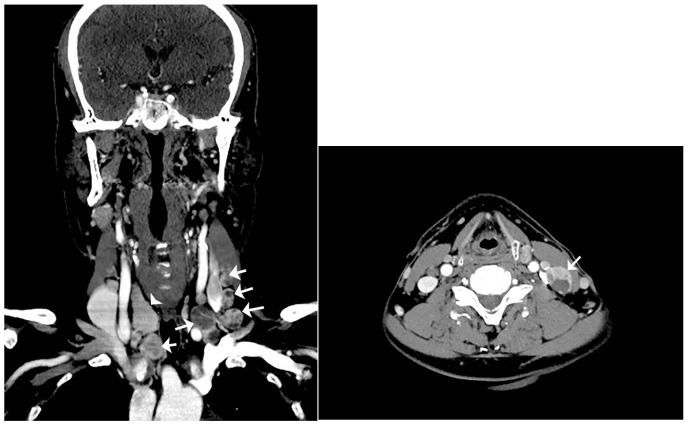
Computerized tomography (CT) scan of the neck. Left: coronal CT scan of the neck reveals multiple enlarged lymph nodes with internal variable cystic change involving bilateral neck of right level III and left levels II, III, and IV, and right lower cervical chain (arrow: multiple enlarged lymph nodes with cystic change; arrowhead: right lobe of thyroid). Right: axial CT scan of the neck reveals an enlarged lymph node with internal cystic change at left level III (arrow: the most enlarged lymph node with cystic change). Therefore, total thyroidectomy and cervical lymph node dissection were performed to distin-guish lymph node metastasis of occult thyroid cancer from seeding of thyroid tissue due to pre-vious thyroid surgery. Resected bilateral cervical lymph nodes were enlarged to the maximum dimensions of 3.5 × 1.7 × 1.0 cm. The cut surface of the lymph node and thyroid gland showed multiple variable-sized pale tan to brown solid nodules with cystic changes (Figure 3).

**Figure 3:**
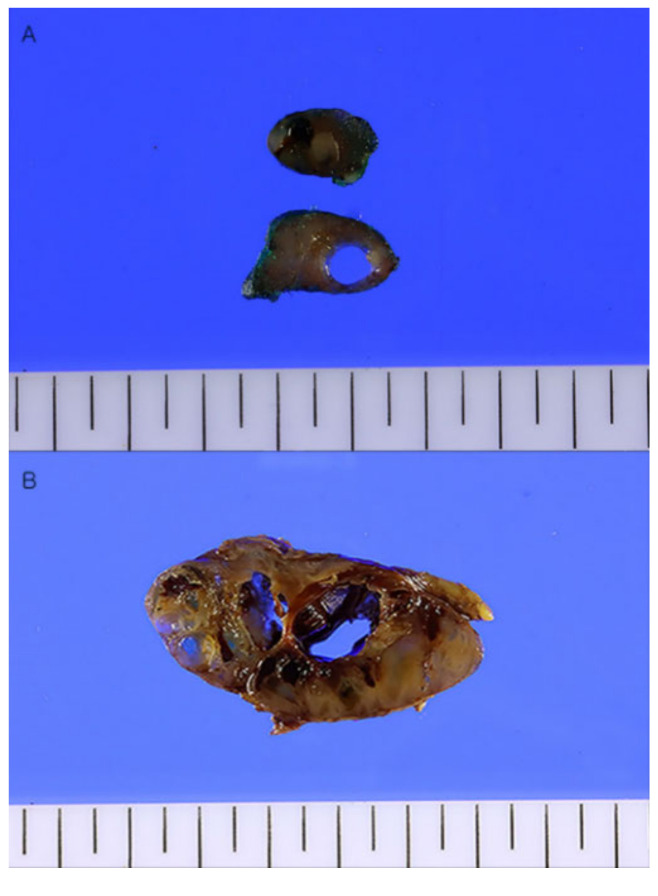
Gross findings of cervical lymph nodes (**A**) and right thyroid gland (**B**). (**A**) The cervical lymph node is enlarged 2.5 cm in maximum length. The cut surface of the lymph node shows a pale tan to brown solid appearance with cystic change. (**B**) The thyroid gland shows multiple variable-sized pale tan to brown solid and cystic nodules. In the areas of the superior thyroid artery and sternothyroid as well as enlarged cervical lymph nodes, benign thyroid tissues showed nodular proliferation with cystic changes. Some enlarged lymph nodes occupied almost the entire area (Figure 4A,B). Both thyroid lobes also had multiple follicular nodules covering almost all areas. Although follicular nuclei showed little irregularity in the nodular hyperplasia of the thyroid gland, atypical malignant cytologic features such as intranuclear grooves, pseudo-inclusions, and ground-glass appearances of nuclei were not observed (Figure 4C), except for an occult papillary microcarcinoma of a follicular variant (0.2 cm in diameter) in the remnant left thyroid (Figure 4E). The occult thyroid microcarcinoma was shown to be positive in the immunohistochemical staining for HBME-1, Galectin-3, and Cytokeratin 19. Among them, HBME-1 showed strong positivity (Figure 4F) compared to the surrounding benign thyroid tissue and nodular hyperplasia (Figure 4D).

**Figure 4:**
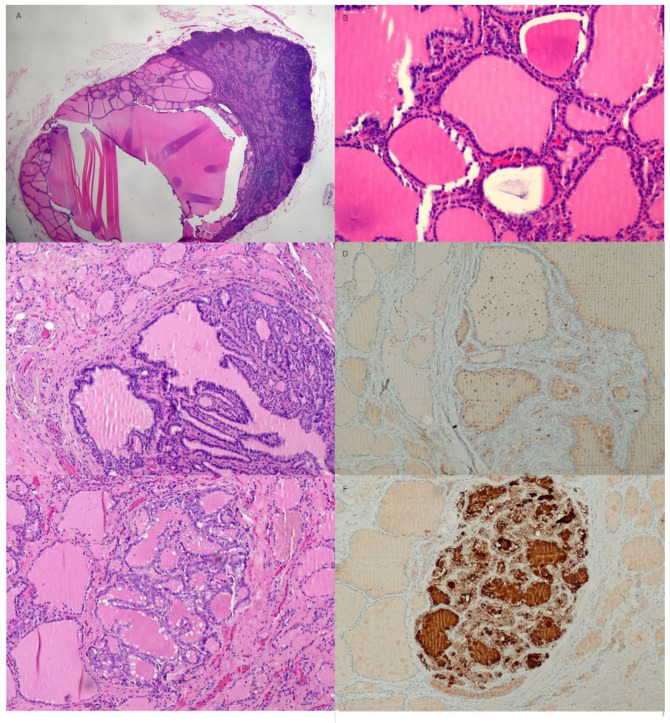
Histologic features of the cervical lymph nodes and thyroid gland. (**A**) A cervical lymph node is replaced by benign thyroid tissue with cystic change (hematoxylin and eosin stain (H&E), X20). (**B**) Numerous thyroid follicles containing pink colloid material are tightly packed (H&E, X200). (**C**) Both lobes of the thyroid gland have multiple follicular nodules showing mild nuclear enlargement and irregularity but no definite evidence of nuclear grooves or intranuclear inclusions indicating papillary thyroid carcinoma (H&E, X100). (**D**) The immunohistochemical stain for HBME-1 shows a negative reaction in the adenomatous nodule of the thyroid (HBME-1, X100). (**E**) A well-demarcated, 2 mm sized, atypical nodule composed of irregular nuclei with a ground-glass appearance in the remnant left lobe of the thyroid (H&E, X100). (**F**) Strong positive HBME-1 staining in the follicular cytoplasms of an atypical thyroid nodule was seen (HBME-1, X100). Additionally, real-time PCR for BRAF V600E mutation did not detect BRAF V600E mutations in any of the nodules of the thyroid and cervical lymph nodes. In case of cervical lymphadenopathy, it is necessary to differentiate between lymph node metastasis of occult thyroid carcinoma and that of benign intranodal thyroid tissue. The probability of clinically detecting occult thyroid carcinoma by autopsy in cervical lymph nodes is over 25% [12]. In addition, the probability of occult thyroid carcinoma metastasizing to the cervical lymph node is 0.7% [13]. For patients who have undergone cervical lymphadenectomy, the probability of having benign thyroid inclusions or psammoma bodies is 0.8%, and the probability of having benign intranodal thyroid tissue in the head and neck lymph nodes based on autopsy or neck dissection is 0.6 to 5.0% [13,14]. Therefore, benign intranodal thyroid tissue can have a false-positive result of metastasis of occult thyroid carcinoma in the cervical lymph node, therefore, a careful approach is needed for preoperative discrimination. In the US, the probability of malignancy is 74.3% in cases of diffusely increased echogenicity, whereas the probability of malignancy is not low at 43.8% for focally increased intranodal echogenic islands. In addition, when features such as intranodal calcification, intranodal cystic changes, and abnormal vascular patterns are observed, lymph node metastasis of papillary thyroid carcinoma can be suspected [14,15]. Additionally, on the cervical CT scan, when strong or heterogenous enhancement, intranodal calcification, or cystic change is observed, malignancy is suspected [14,16]. In addition to the typical nuclear features of papillary thyroid carcinoma with intranuclear grooves, intranuclear inclusion bodies and a ground-glass appearance, immunohistochemical staining, especially HBME-1, is helpful in distinguishing between intranodal benign thyroid tissue and metastatic papillary carcinoma in the lymph nodes [9]. Additionally, real-time PCR for BRAF V600E mutation test can be performed to further enhance the accuracy of the diagnosis. There are several hypotheses regarding the origin of benign intranodal thyroid tissue. It is believed to be due to abnormal migration of benign tissue during embryogenesis, neoplastic transformation of the remaining cells, or metastatic spread of a clinically silent malignancy [14]. In this case, considering that the patient had benign thyroid tissue in the cervical lymph nodes ranging from level II to level VI, the benign thyroid tissue appeared to be embedded in the chain of cervical lymph nodes because the benign thyroid tissue was adherent to the surrounding tissue from previous thyroid surgery and deep cervical inflammation. In the embedded benign thyroid tissue of cervical lymph nodes, cystic changes were commonly observed. In conclusion, benign thyroid tissue could be included in many lymph nodes along the cervical lymph node chain because of adhesions from previous thyroid surgery and deep cervical inflammation. Benign intranodal thyroid tissues may enlarge with cystic changes. Therefore, it is necessary to differentiate it from metastatic lymph nodes of thyroid papillary carcinoma. Immunohistochemical staining for HBME-1 and real-time PCR for the BRAF V600E mutation, in addition to histologic examination, are also helpful.

## Data Availability

Not applicable.
